# Muscle-derived miR-34a increases with age in circulating extracellular vesicles and induces senescence of bone marrow stem cells

**DOI:** 10.18632/aging.101874

**Published:** 2019-03-25

**Authors:** Sadanand Fulzele, Bharati Mendhe, Andrew Khayrullin, Maribeth Johnson, Helen Kaiser, Yutao Liu, Carlos M. Isales, Mark W. Hamrick

**Affiliations:** 1 Medical College of Georgia, Augusta University, Augusta, GA 30912, USA

**Keywords:** exosomes, senescence, muscle-bone crosstalk, sarcopenia, osteoporosis

## Abstract

Extracellular vesicles (EVs) are known to play important roles in cell-cell communication. Here we investigated the role of muscle-derived EVs and their microRNAs in the loss of bone stem cell populations with age. Aging in male and female C57BL6 mice was associated with a significant increase in expression of the senescence-associated microRNA miR-34a-5p (miR-34a) in skeletal muscle and in serum –derived EVs. Muscle-derived, alpha-sarcoglycan positive, EVs isolated from serum samples also showed a significant increase in miR-34a with age. EVs were isolated from conditioned medium of C2C12 mouse myoblasts and primary human myotubes after cells were treated with hydrogen peroxide to simulate oxidative stress. These EVs were shown to have elevated levels of miR-34a, and these EVs decreased viability of bone marrow mesenchymal (stromal) cells (BMSCs) and increased BMSC senescence. A lentiviral vector system was used to overexpress miR-34a in C2C12 cells, and EVs isolated from these transfected cells were observed to home to bone *in vivo* and to induce senescence and decrease Sirt1 expression of primary bone marrow cells *ex vivo*. These findings suggest that aged skeletal muscle is a potential source of circulating, senescence-associated EVs that may directly impact stem cell populations in tissues such as bone via their microRNA cargo.

## Introduction

Extracellular vesicles (EVs), including exosomes and microvesicles, are now acknowledged to play important roles in a number of disease processes such as cancer metastasis and neurodegeneration [1,2]. The potential for EVs to transport cargo of microRNAs (miRNAs), lipids and proteins between cell populations suggests that these membrane-bound particles may be involved in crosstalk among different organ systems [3,4]. Such crosstalk is likely to underlie degenerative changes that can occur in multiple organ systems with aging and disease. For example, adipose tissue is recognized as a secretagogue for a number of inflammatory cytokines that can negatively impact multiple tissues [5], and muscle is a source of myokines that can positively impact various organs and tissues following exercise [6]. How EVs fit into this picture is not entirely clear, but EVs have been isolated from both adipocytes [7] and myotubes [8] suggesting that they may be able to function in a manner similar to adipokines and myokines, respectively. In support of this idea muscle-derived EVs have been detected in human serum, and their miRNA cargo is altered with physical activity [9].

Recently it has been discovered that cells undergoing senescence may secrete factors that can impact neighboring cells and tissues, factors collectively referred to as the senescence-associated secretory phenotype or SASP [10]. Most studies to date on the SASP have focused on inflammatory factors that are secreted by senescent cells such as IL-6 and IL-1β. It has, however, been proposed that EVs may be part of the SASP, and EVs have been identified that are secreted from senescent cells such as those cells in senescent lung [11]. The SASP is linked to age-related organ dysfunction since aging is associated with an accumulation of senescent cells. Yet the specific SASP-related factors carried by EVs with aging have not yet been defined, and their role in tissue crosstalk has not yet been explored. Aging is known to be accompanied by loss of muscle mass, a phenomenon referred to as sarcopenia [12,13]. Here we characterize EVs released from aged muscle *in vivo*, and the miRNAs secreted by muscle cells in response to age-associated stimuli *in vitro*. We show that muscle-derived EVs carrying senescence-associated miRNAs can induce cellular senescence in bone stem cells, revealing a potential mode of inter-organ crosstalk that may contribute directly to tissue dysfunction with aging.

## RESULTS

### miR-34a expression is significantly increased with age in mouse skeletal muscle and in serum extracellular vesicles

**Figure 1:**
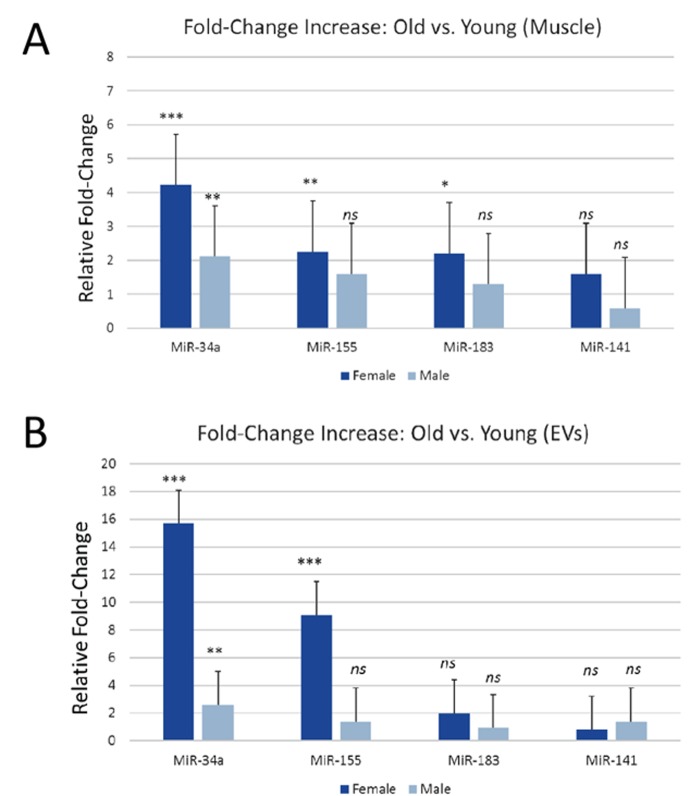
**miR-34a is increased in mouse skeletal muscle and in serum EVs with age.** (**A**) Expression levels of miR-34a, miR-155, miR-183, and miR-141 in skeletal muscle of young and aged male and female mice. (**B**) Expression levels of miR-34a, miR-155, miR-183, and miR-141 in serum EVs from young and aged male and female mice. *P<.05, **P<.01, ***P<.001.

Aging, inflammation, and oxidative stress are associated with increases in several miRNAs including miR-34a, miR-141, miR-155, and miR-183 which in turn target genes involved in cell survival such as Bcl2, Sirt1, FGF7, and Foxo1 among others [14–18]. We therefore analyzed expression of these miRNAs in skeletal muscle in serum EVs derived from young and aged male and female mice. Skeletal muscle from male and female aged mice showed a significant increase in miR-34a, whereas miR-141 was not significantly increased in male or female muscle and miR-155 and miR-183 were only increased in muscle from female mice (Figure 1A). EVs from serum of aged mice showed a significant increase in miR-34a in both males and females, whereas miR-155 was only increased in EVs from female mice (Figure 1B). EVs from male and female mice showed no marked increase in miR-183 or miR-141. Bivariate correlations indicate a significant (P<.001) association between muscle and serum EV miR-34a fold-change values (r=.66).

### Muscle-derived extracellular vesicles show increased miR-34a expression with age

**Figure 2:**
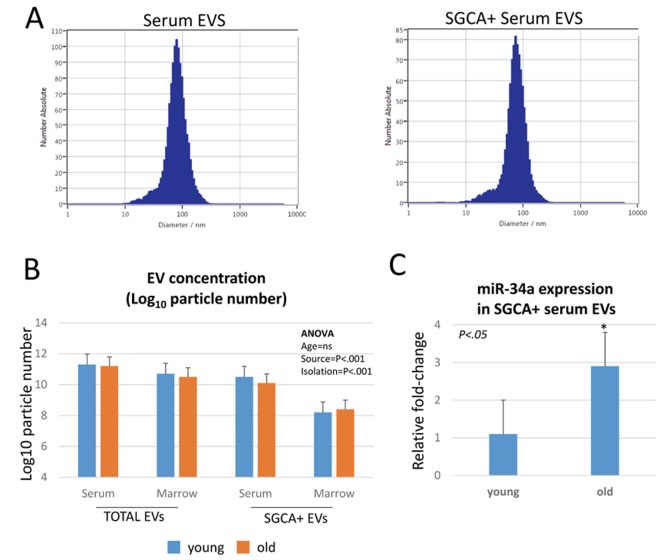
**miR-34a is increased with age in muscle-derived, alpha-sarcoglycan positive (SGCA+) EVs**. (**A**) Representative particle distribution histograms from nanoparticle tracking analysis showing similar particle sizes between serum EVs and SGCA+ serum EVs. (**B**) Particle concentrations of SGCA+ EVs are significantly lower than total serum EVs, and SGCA+ EVs in bone marrow are significantly less abundant than SGCA+ EVs in serum. (**C**) SGCA+ EVs show a significant increase in miR-34a expression with age.

We isolated EVs from serum in young and aged mice and then used immunoprecipitation for alpha-sarcoglycan (SGCA) to isolate muscle-derived EVs [9]. Representative particle distributions from nanoparticle tracking data show that particle sizes are similar between serum-derived EVs and SGCA+ EVs, and the particle diameters are less than <100 nm consistent with the known size of exosomes (Figure 2A). Nanoparticle tracking data indicate that SGCA+ EVs represent slightly less than 10% of EVs in serum (Figure 2B), consistent with previous findings using human serum [9]. SGCA+ EVs are significantly less abundant in bone marrow interstitial fluid compared to serum (Figure 2B). ANOVAs show no significant age effect on EV concentration, and there is a significant interaction between EV source and isolation approach, with SGCA+ EVs being significantly lower in marrow than in serum (Table 1). Real-time PCR data on SGCA+ EVs from serum indicate that miR-34a is significantly increased in SGCA+ EVs from aged mice compared to young mice (Figure 2C).

**Table 1:** ANOVA results from nanoparticle tracking analysis of EV concentration (log-transformed values. Note significant interaction between source (serum or marrow) and isolation technique (normal or SGCA+).

Source	Type III Sum of Squares	df	Mean Square	F	Sig.
Corrected Model	172.153^a^	7	24.593	10.496	.000
Intercept	189.328	1	189.328	80.799	.000
AGE	2.532	1	2.532	1.080	.306
SOURCE	45.066	1	45.066	19.233	.000
ISOLATION	92.315	1	92.315	39.397	.000
AGE * SOURCE	2.403	1	2.403	1.026	.318
AGE * ISOLATION	1.974	1	1.974	.843	.365
SOURCE * ISOLATION	28.930	1	28.930	12.346	.001
AGE * SOURCE * ISOLATION	.202	1	.202	.086	.771
Error	82.012	35	2.343		
Total	390.383	43			
Corrected Total	254.165	42			

a. R Squared = .677 (Adjusted R Squared = .613)

### Oxidative stress increases miR-34a in myoblast- and myotube-derived EVs, and these EVs can induce cellular senescence in bone stem cells

**Figure 3:**
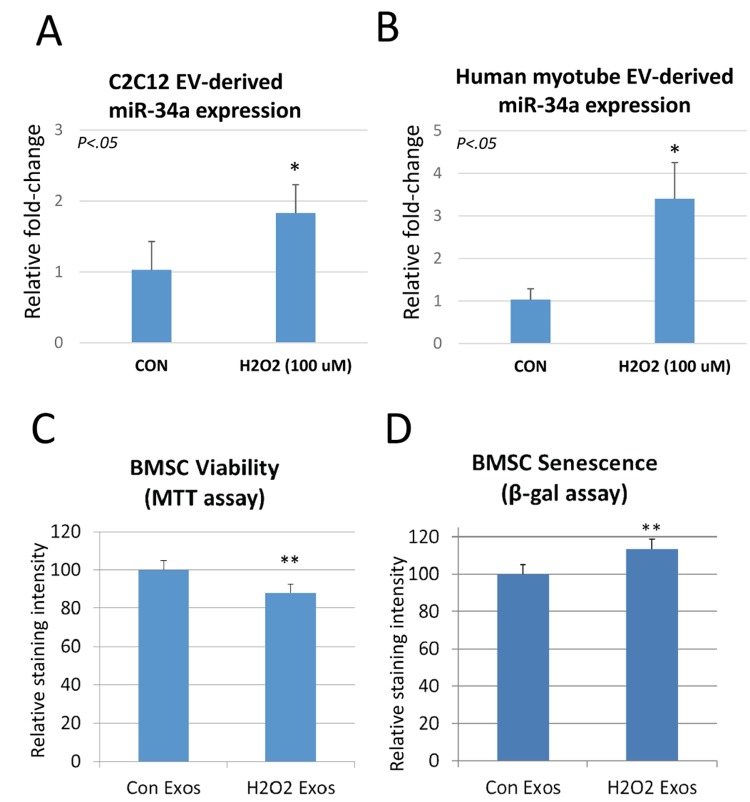
**Hydrogen peroxide increases miR-34a in EVs secreted by C2C12 myoblasts and human myotubes, and these EVs can reduce bone stem cell (BMSC) viability and increase senescence**. (**A**) EVs isolated from C2C12 cells treated with hydrogen peroxide show a significant increase in miR-34a. (**B**) EVs isolated from human myotubes treated with hydrogen peroxide show a significant increase in miR-34a. (**C**) BMSC viability indicated by MTT assay is significantly reduced after treatment with EVs isolated from these C2C12 cells exposed to hydrogen peroxide. (**D**) BMSC senescence measured by beta-galactosidase (β-gal) assay is significantly increased after treatment with EVs isolated from these C2C12 cells exposed to hydrogen peroxide. *P<.05, **P<.01.

Reactive oxygen species are increased in skeletal muscle with aging, and as shown above muscles from aged mice show elevated levels of miR-34a and muscle-derived EVs from aged mice also have increased levels of miR-34a. We tested the hypothesis that oxidative stress might increase the production of EVs enriched in miR-34a *in vitro* by treating mouse C2C12 myoblasts and primary human myotubes with hydrogen peroxide. Hydrogen peroxide significantly increased levels of miR-34a in EVs isolated from the conditioned medium (Figure 3A, B). We then analyzed the bioactivity of these EVs by treating bone marrow mesenchymal (stromal) cells (BMSCs) from young adult mice. We utilized BMSCs for this system because aging is characterized by significant reduction in the population of BMSCs [19]. EVs from C2C12 cells exposed to hydrogen peroxide significantly decreased cell viability (Figure 3C) and increased cellular senescence (Figure 3D) compared to EVs from C2C12 cells not exposed to hydrogen peroxide.

### EVs from cells overexpressing miR-34a can decrease BMSC viability and increase cellular senescence

**Figure 4:**
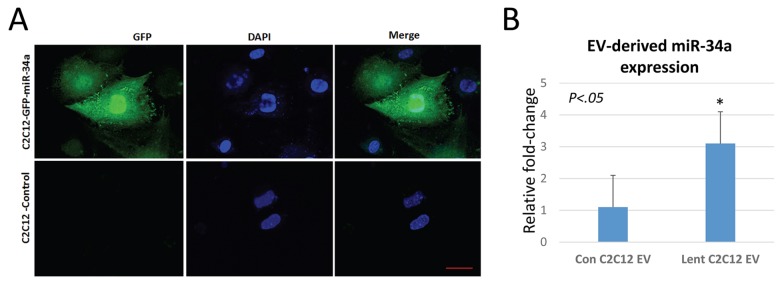
**C2C12 cells overexpressing miR-34a secrete EVs with elevated levels of miR34a**. (**A**) Confocal images of C2C12 cells transfected with a lentivirus overexpressing miR-34a. The virus contains a GFP reporter under a constitutive CMV promoter. Images show GFP expression in transfected cells. Blue staining represents nuclear DAPI staining. Scale bar = 20 µm. (**B**) Analysis of miR-34a expression in EVs from transfected and non-transfected cells shows a three-fold increase in miR-34a in EVs isolated from conditioned medium of transfected cells.

**Figure 5:**
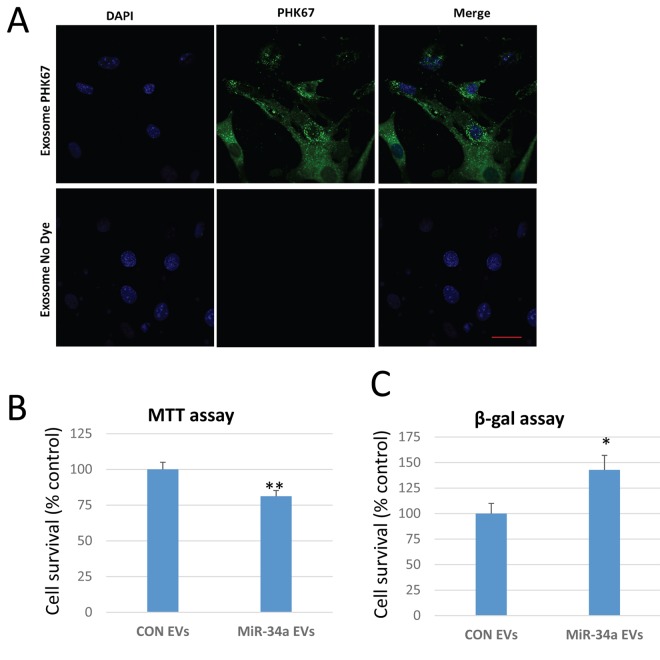
**EVs from C2C12 cells overexpressing miR-34a reduce BMSC viability and increase senescence.** (**A**) Confocal images of BMSCs treated with EVs isolated from conditioned medium of C2C12 cells overexpressing miR-34a. EVs are unlabeled (control, bottom row) or labeled with the membrane dye PKH67 (top row). Images show abundant EVs in cytoplasm of BMSCs. Blue staining represents nuclear DAPI staining. Scale bar = 20 µm. (**B**) BMSC viability indicated by MTT assay is significantly reduced after treatment with EVs isolated from C2C12 cells overexpressing miR-34a. (**C**) BMSC senescence measured by beta-galactosidase (β-gal) assay is significantly increased after treatment with EVs isolated from these C2C12 cells overexpressing miR-34a. *P<.05, **P<.01.

We utilized a lentiviral vector system to overexpress miR-34a in C2C12 cells tagged to green-fluorescent protein. Validation of the system using confocal imaging shows that miR-34a is highly expressed in cells transfected with the lentivirus (Figure 4A). EVs isolated from conditioned medium of C2C12 cells overexpressing miR-34a show a three-fold increase in miR-34a compared to EVs from untransfected cells (Figure 4B). Confocal imaging of BMSCs treated with PKH67-labeled EVs from C2C12 cells overexpressing miR-34a show that BMSCs readily take up these labeled vesicles (Figure 5A). Quantitative analysis of BMSC viability and senescence shows that EVs from miR-34a overexpressing C2C12 cells significantly decrease cell viability and increase cellular senescence (Figure 5B, C).

### EVs from cells overexpressing miR-34a home to bone marrow *in vivo* and decrease Sirt1 expression *ex vivo*

**Figure 6:**
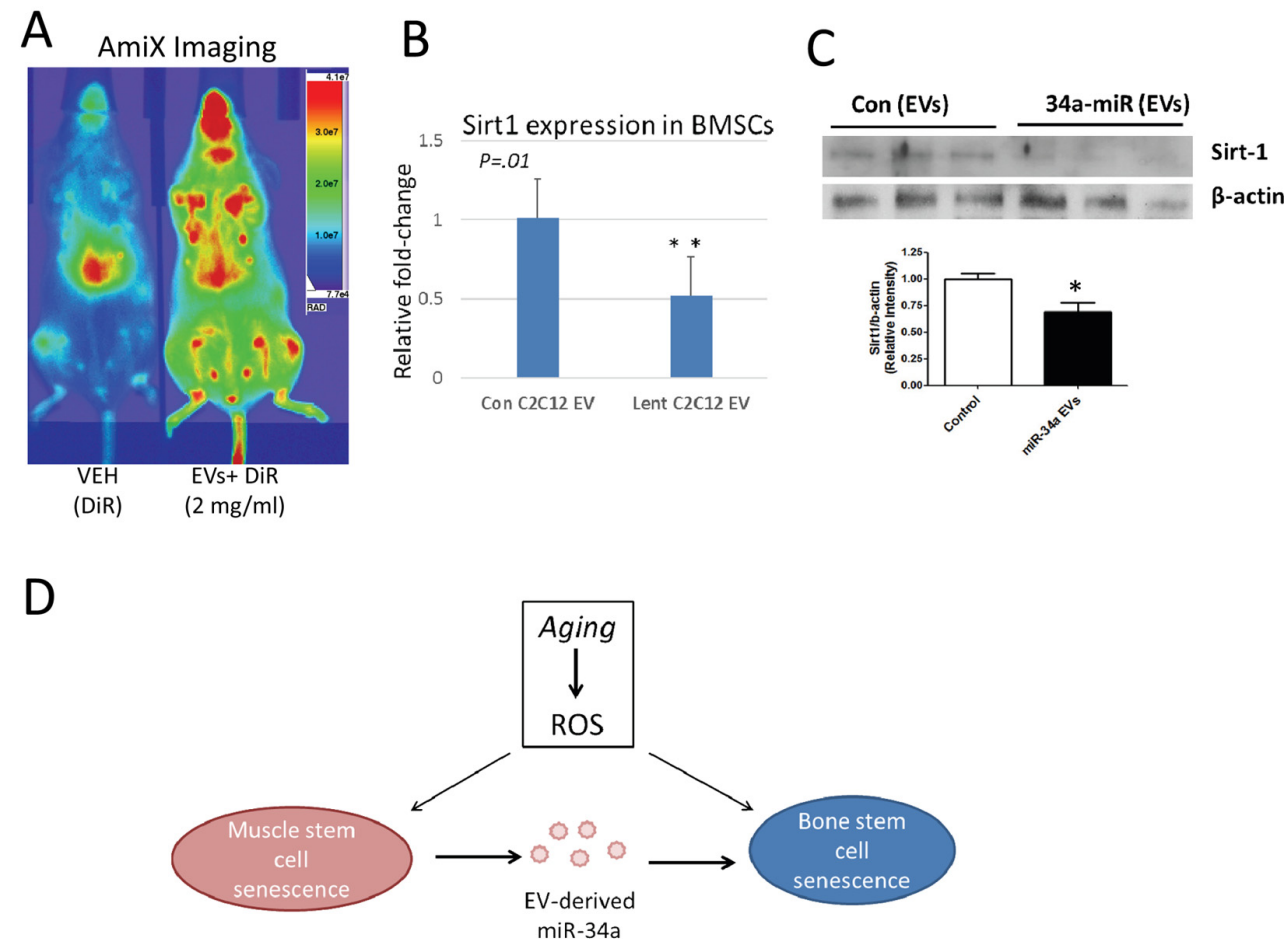
**EVs from C2C12 cells overexpressing miR-34a home to bone marrow *in vivo* and reduce Sirt1 expression *ex vivo*.** (**A**) Mice were injected via tail vein with DiR dye alone (VEH) or EVs from C2C12 cells overexpressing miR-34a labeled with DiR (EVs + DiR) and imaged with AmiX imaging. Mice receiving labeled EVs show high image intensity in the metaphyseal regions of long bones. (**B**) Bone marrow cells flushed from untreated mice and cultured in the presence of EVs from miR-34a overexpression cells show reduced Sirt1 expression compared to cells cultured with EVs from control C2C12 cells. (**C**) Bone marrow cells flushed from untreated mice and cultured in the presence of EVs from miR-34a overexpression cells show reduced Sirt1 protein compared to cells cultured with EVs from control C2C12 cells. Top image is from protein of adherent cells, graph includes pooled data from both adherent and non-adherent cells. *P<.05, **P<.01. (**D**) Working model summarizing changes in muscle and bone with age, and the role of EV-derived miR-34a in muscle and bone senescence. ROS = reactive oxygen species.

Previous work suggests that EVs derived from C2C12 cells can travel to a variety of organs *in vivo*, including the limb [20]. We tested the hypothesis that EVs enriched in miR-34a may reach the bone marrow microenvironment *in vivo* by labeling these EVs with the infrared dye DiR and then imaging mice 24 hours after tail vein injection. Images show that DiR-labeled vesicles are detected in both the fore- and hindlimb (Figure 6A). We flushed primary bone marrow cells from the limb bones of untreated mice and cultured these cells *ex vivo* in the presence of EVs derived from miR-34a overexpressing cells or with EVs from untransfected C2C12 cells. We then analyzed expression for Sirt1, since miR-34a is known to target Sirt1 in BMSCs [18] and Sirt1 plays a key role in cell survival [21]. Analysis of mRNA levels and protein show that EVs from miR-34a overexpressing C2C12 significantly reduced Sirt1 mRNA and protein in primary bone marrow cells (Figure 6B, C).

## DISCUSSION

Previous work suggests that extracellular vesicles may play important roles in the aging process. Changes in circulating EV cargo, such as miRNAs, proteins, and lipids characterize older versus younger populations, and many of these alterations in EV cargo are thought to reflect cell- and tissue-level changes in response to age-associated stimuli such as inflammation and oxidative stress [22–25]. Our work provides additional support for this concept, and indicates that elevated oxidative stress in skeletal muscle with age is associated with changes in the miRNA cargo of muscle-derived EVs. The *in vivo* data are corroborated by *in vitro* experiments showing the exposure of muscle cells to oxidative stress significantly alters the miRNA profile of secreted EVs. These findings are consistent with previous work demonstrating that myoblasts and myotubes actively secrete EVs *in vitro* [8,26–29], that muscle-derived EVs can be detected in the circulation *in vivo* [9], and that the cargo of muscle-derived EVs is altered by changes in the systemic environment [4,9].

We evaluated age-associated changes in several miRNAs with aging, and found that miR-34a-5p is significantly increased in muscle and in muscle-derived EVs with aging and with myoblast exposure to oxidative stress. These results support previous findings that miR-34a is increased with aging in the circulation [30] and in skeletal muscle [31], and that its elevated expression is associated with muscle atrophy and myopathy [31,32]. miR-34a is regulated at least in part by p53 [18] and its increased expression with aging is consistent with a role in for miR-34a in cell death. This role for miR-34a is further indicated by the capacity of miR-34a to promote DNA damage and inhibit DNA repair [33]. miR-34a is known to induce senescence in vascular smooth muscle cells and cardiomyocytes and to increase cardiac fibrosis [34,35]. miR-34a knockout attenuates cognitive deficits in APP/PS1 mice [36] and calorie restriction promotes cell survival in the mouse brain by downregulating miR-34a [37]. Together, these studies combined with our new data support the notion that increased miR-34a expression with aging in skeletal muscle is likely associated with elevated muscle damage. It is thought that a primary mechanism by which miR-34a mediates senescence and cell survival is by suppressing the pro-survival deacetylase Sirt1. Sirt1 is downregulated and miR-34a increased in various cell types with aging [38], Sirt1 is a well-established target of miR-34a [18], and decreased Sirt1 activity in muscle with aging is associated with impaired muscle performance [39]. Interestingly we also observed sex differences in miR-34a expression, with females showing a greater increase in miR-34a expression with age than males. A similar difference has been noted in mouse cardiac tissue, with females showing greater miR-34a expression than males [40]. There are well-documented sex differences in microRNA expression in a variety of cells and tissues [41], and in mice females show a more marked decline in grip strength and muscle absolute force with aging than male mice [42,43]. These sex-specific differences may explain the greater miR-34a expression with aging that we observed in female mouse muscle and in EVs.

It is well-known that crosstalk exists between muscle and bone, and that muscle can secrete a number of factors that can promote bone formation and suppress bone resorption [6,44,45]. Sirt1 has positive effects on bone mass through its activation of the osteoblast transcription factor Runx2 and its inhibition of NF-κB signaling [46,47]. miR-34a has detrimental effects on bone by suppressing bone formation by osteoblasts [48] and by decreasing survival of BMSCs [21,49] which are precursors of osteoblasts. Our data are consistent with these studies and indicate that muscle-derived EVs carrying miR-34a can reduce Sirt1 expression in BMSCs and also induce BMSC senescence. These results may represent a novel pathway by which muscle can influence bone physiology (Figure 6D). The majority of studies to date on muscle-bone crosstalk have revealed positive effects of muscle-derived factors on bone [44,45]. The data presented here suggest that the accumulation of reactive oxygen species in skeletal muscle with aging can potentially have negative effects on bone due to circulating, muscle-derived EVs carrying miR-34a (Figure 6D). Future studies may be directed at identifying other factors carried by muscle-derived EVs and determining their effects on various organs and tissues such as bone, liver, and adipose tissue. miR34a can target other factors important for muscle and bone repair and regeneration such as Notch, Jagged1 and Numb [18], and it is possible that the negative, age-associated effects of EV-derived miR-34a may be mediated by suppression of these factors in addition to Sirt1.

The *in vivo* and *in vitro* experiments performed here indicate that hydrogen peroxide stimulates and increase in EV-derived miR-34a. Previous studies have shown that, in general, overexpression of a particular miRNA in a cell is linked with greater abundance of that miRNA in secreted EVs [50]. A limitation of our study is that we have not yet performed mechanistic analyses linking ROS exposure to EV packaging and secretion. We hypothesize that the release of specific miRNAs within EVs is a mechanism for cell survival. Previous authors have proposed that EV release is a process that removes harmful materials from the cell cytoplasm such as accumulated nuclear DNA or lipids such as ceramides [51,52]. We expect that cellular stressors that occur with aging, such as exposure to inflammatory factors and/or reactive oxygen species, increase miR-34a expression as a by-product of p53 activation. This is known to occur in the setting of sepsis, injury and inflammation [53]. Removal of stress- and senescence-associated miRNAs such as miR-34a via extracellular vesicle secretion would be a mechanism to promote cell survival; however, it is likely that increasing the circulating concentration of such “inflammiRs” will ultimately have detrimental effects on neighboring cells and tissues by delivering these inflammiRs via EV membrane fusion and endocytosis. Interventions such as physical activity and dietary restriction may ultimately reduce the systemic burden of these stress- and senescence-associated miRNAs carried within circulating EVs and in turn reduce cell- and tissue-level damage with aging.

## MATERIALS AND METHODS

### Sample collection and preparation from young and old mice

C57BL/6 mice were obtained from the National Institute on Aging at 6 and 24 months of age, approximately 5-6 males and 5-6 females per age group. Mice were euthanized by CO_2_ overdose followed by thoracotomy as approved by the Augusta University IACUC. Tibialis anterior muscles were dissected free and snap frozen in liquid nitrogen for miRNA isolation using the miRNeasy kit (Qiagen) following manufacturer specifications. Blood was collected via cardiac puncture and serum isolated by clotting for 1 hr and then centrifugation for 15 min at 5,000 rpm.

### EV isolation and nanoparticle tracking analysis

**Figure 7:**
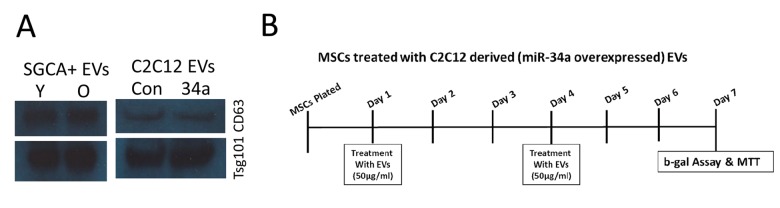
**Western blot for exosome markers and experimental design for in vivo treatment.** (**A**) SGCA+ EVs isolated from serum of young (Y) and old (O) mice as well as from conditioned medium of normal C2C12 cells and cells overexpressing miR-34a are positive for the exosome markers CD63 and TSG101. (**B**) Timeline, EV doses, and outcome measures for *in vitro* BMSC treatments.

EVs were isolated from serum and flushed bone marrow interstitial fluid from the femur [19] using 8% PEG as described by Rider and colleagues [54]. Particle size and concentration were quantified using the Zetaview instrument from Particle Matrix [55,56]. Immunoprecipitation for muscle-derived EVs was performed using an antibody specific for muscle alpha-sarcoglycan (SGCA) following ref [9]. In brief, we used the Expedeon Lightning-Link® Rapid Biotin – Type B kit to label the SGCA monoclonal antibody (IVD3(1)A9) from the Developmental Studies Hybridoma Bank. Immunoselection was performed using the Pierce™ Streptavidin Plus UltraLink™ Resin kit following manufacturer specifications. The EVs were initially mixed with prepared streptavidin resin for an hour to remove any nonspecific binding elements. EVs were removed and mixed with pretreated biotinylated SGCA-streptavidin resin for overnight incubation. After incubation the sample was spun down and supernatant removed. The resin was then treated with 0.1M glycine-HCl (pH 2.5-3.0) and supernatant collected after spin down and acidic conditions neutralized with Tris-HCL (pH 8.0). miRNAs were isolated from serum EVs as well as from SGCA+ serum EVs. Western blot for the exosomal markers CD63 (Santa Cruz #sc-15363) and TSG101 (Santa Cruz #sc-7964) was performed following standard procedures (Figure 7A). We have submitted all relevant data of our experiments to the EV-TRACK knowledge base (EV-TRACK ID: EV180077; http://evtrack.org/).

### Analysis of miRNA expression in skeletal muscle and EVs

The concentration of miRNA from skeletal muscle samples and EVs was determined using a NanoDrop spectrophotometer (Thermo Scientific). Specifically, we analyzed expression of miR-34a-5p, miR-155-5p, miR-183-5p, and miR-141-3p using primers from Qiagen. Two hundred nanograms of enriched miRNAs were converted into cDNA using miScript II RT Kit (from Qiagen). Fifty pictograms of cDNA were amplified in each qRT-PCR using SYBR Green I and miR-specific primers (Qiagen, Valencia, CA, USA). The real-time qRT-PCR was performed on a Bio-Rad MyiQ machine with following cycling parameters: 95 °C for 10 mins, then 40 cycles of 95 °C for 15 s, 60 °C for 30 s and 72 °C for 30 s. The average of RNU6 and SNORD was used as normalization reference genes for miRs. Relative expression of miRNA was evaluated by using the comparative CT method (ΔΔCt).

### *In vitro* studies utilizing C2C12 myoblasts overexpressing miR-34a

We used a lentiviral system to overexpress miR-34a in mouse C2C12 myoblasts as a mechanism to produce exosomes enriched in miR-34a. The lentiviral particles were purchased from Genecopoeia (PP-MmiR3342-MR03), Polybrene® (sc-134220) and puromycin (sc-108071) was purchased from Santa Cruz Biotechnology, Inc. USA. In brief, C2C12 cells were plated at 30–50% confluence and transfected with appropriate dilutions of lentivirus particles and polybrene. Forty-eight hours after transfection, the cells were cultured in growth medium containing puromycin (2 μg/ml) to obtain the stable, transfected C2C12 cells. The efficiency of overexpression of miR-34a in C2C12 cells EVs was analyzed by fluorescence imaging real-time PCR (Figure 4). Primary mouse BMSCs were isolated from young adult mice as previously described [19,57]. BMSCs were treated with EVs (50 µg/ml) isolated from normal C2C12 cells or cells overexpressing miR-34a at day 1 and day 4 of culture (Figure 7B). At day 7 cells were analyzed using MTT assay (Fisher) to assess cell viability and β-galactosidase assay (Enzo Life Sciences) to measure cellular senescence following manufacturer specifications. Uptake of EVs was verified using PKH67 labeling of EVs followed by confocal imaging.

### In vitro studies utilizing EVs from C2C12 cells and human myotubes exposed to hydrogen peroxide

C2C12 cells were prepared as described above, treated with H_2_O_2_ (100µM) for 24hrs, and EVs isolated as described for cells overexpressing miR-34a. C2C12 (ATCC® CRL-1772™) and Human Skeletal Myoblast (HSkM) cells (Gibco, catalog#A1140) were grown to 80% confluence in growth media for 2 days followed by myotube differentiation. Myotube differentiation is initiated upon reaching confluence by switching the cells to medium containing 2% horse serum for 5-6 days. Then, cells were treated with hydrogen peroxide (H_2_O_2_) in 1% exo-free media for 24hrs. Exosomes were isolated from cell culture supernatants as per published method. BMSCs cells were plated separately for MTT assay and β-galactosidase assay at an initial density of 3000/cm^2^ in 96-well plates using 5% FBS to support overnight attachment. The following day, cells were treated with control and H_2_O_2_ derived EVs (50μg/ml) separately with 1% FBS (exosome free) media for 48 hrs. MTT and β-galactosidase assays were performed as described above.

### *In vivo* tracking of C2C12-derived EVs and *ex vivo* treatment of bone marrow cells

EVs isolated from conditioned medium of C2C12 cells were labeled labeled with the membrane dye DiR (Xenolight, Perkin Elmer) following manufacturers recommendations. DiR is a near-infrared lipophilic dye that binds the lipid bilayer surrounding EVs [20]. Its emission spectra is only visible using infrared CCD imaging. We injected 100 µl of DiR dye alone or DiR-labeled EVs at 1.0 mg/ml and then imaged (Ex =710, Em=790) 24 hrs later using an Ami X spectral imaging instrument. Bone marrow cells were flushed from long bones of young adult mice and adherent and non-adherent cells cultured and treated (50µg/ml EVs) with normal C2C12 EVs and miR-34a overexpressed EVs for 18 hrs. The cell lysate was prepared for western blot analysis as per our published method [57]. In brief, protein was extracted from cell culture lysate, subjected to SDS-PAGE, and transferred to nitrocellulose membranes. Membranes were incubated with a polyclonal antibody against Sirt1 (Millipore Anti-Sirt1 Cat # 07-131), and beta-actin (Santa Cruz Biotechnology, Santa Cruz, CA) overnight at 4 ºC, followed by incubation with appropriate secondary antibody. Proteins were visualized with an ECL Western blot detection system (Thermo Scientific, Waltham, MA).

### Statistical analysis

ANOVA was used to compare fold changes in miRNAs between and among different age groups (young vs old), genders (male vs female), and source (muscle vs EVs). Students t-tests were performed for in vitro experiments using treated vs control designs.
